# Protocell Dynamics: Modelling Growth and Division of Lipid Vesicles Driven by an Autocatalytic Reaction

**DOI:** 10.3390/life15050724

**Published:** 2025-04-29

**Authors:** Japraj Taneja, Paul G. Higgs

**Affiliations:** 1 Department of Biochemistry and Biochemical Sciences, McMaster University, Hamilton, ON L8S 4K1, Canada; tanejj1@mcmaster.ca; 2 Department of Physics and Astronomy, McMaster University, Hamilton, ON L8S 4K1, Canada

**Keywords:** protocells, metabolism, lipid vesicles, cell division

## Abstract

We study a computational model of a protocell, in which an autocatalytic reaction sustains itself inside a lipid vesicle. The autocatalytic reaction drives volume growth via osmosis. Membrane area grows due to addition of lipids from the environment. The membrane growth rate depends on the external lipid concentration and on the tension in the membrane. In the absence of division, a cell either reaches a state of homeostasis or grows to a point where the internal reaction collapses. If a cell becomes elongated, it can divide into two smaller spherical vesicles, conserving the total volume and area. We determine when it is energetically favorable for a large vesicle to divide. Division requires the buildup of a difference between the lipid areas on the outer and inner leaflets of the membrane. Division occurs most easily when the rate of flipping of lipids between leaflets is relatively slow. If the flipping is too fast, the parent cell grows large without dividing. There is a typical size at which division occurs, producing two daughter cells of unequal sizes. The smaller and larger daughters regrow to the same typical size before the next division. Protocells with an active metabolism reach a stable state where the internal autocatalytic reaction and the membrane growth are well balanced. Active protocells can grow and divide in conditions where an inactive vesicle without an internal reaction cannot.

## 1. Introduction

A protocell is a simple cell-like structure that may represent the earliest forms of life and the precursors to modern cells. Ganti’s chemoton is often seen as a description of how a protocell must have operated [1]. The chemoton consists of three parts—a metabolism, a replicating genetic system, and a membrane system. In modern cells, these three parts are all mutually dependent. Here, we are interested in how the first protocells might have functioned, and it seems too improbable to require these three systems to be mutually dependent from the beginning.

The question is simpler if we assume that a supply of membrane-forming lipids was available from prebiotic chemistry. In this case, the first protocells could use lipids from the environment without synthesizing them internally. Simple lipids such as fatty acids are likely to have been available on prebiotic Earth [2,3,4]. These can spontaneously form membranes in certain conditions of pH and temperature [5,6,7]. Hence, it is likely that early protocells were housed in vesicles made of simple lipids [8,9]

In order to make a vesicle into a protocell, there must be an autocatalytic reaction inside. We envisage a supply of food molecules from the environment that enter through the lipid membrane. Catalysts inside the cell convert food molecules into more catalysts. Side reactions create waste products that exit the cell. The inside of the cell is in an active non-equilibrium state with a continual turnover of material—a metabolism. When an active state exists in the cell, the total concentration of reagents may be higher inside than outside, in which case osmotic pressure drives the increase in cell volume. If there is a sufficient influx of food through the membrane, the internal reaction is maintained as the cell grows. If lipids are available in the environment, the cell membrane area also grows, and if the area grows sufficiently, the cell can divide into two daughter cells. We wish to determine under which conditions the continued growth and division of a protocell are possible.

We recently considered reaction systems in vesicles and asked which properties they must have to constitute a metabolism [10]. An obvious but non-trivial requirement is that the metabolism must happen in the cell but it must not happen in the environment, otherwise the food molecules would be consumed in the environment and there would be no difference between the inside and outside of the cell. We call this property inside–outside stability (IO-stability). When the reaction system is second-order in the catalyst concentration, there are active and inactive stable states of the same reaction system, and the cell can be IO-stable. When the reaction system is first-order, there is only one stable state; hence, the inside can only be in an active state when the outside is also active, which is not what we need for a protocell.

We have given theoretical examples of reaction networks of types that are IO-stable and types that are not [10]. Metabolism-first theories for the origin of life argue that a small-molecule autocatalytic system existed prior to the origins of replicating molecules such as nucleic acids. However, there are very few examples of real chemical reaction systems that have the required properties. The formose reaction is relevant for the prebiotic synthesis of sugars and is autocatalytic. It has been proposed as a means of sustaining protocells [11] and has also been studied in droplet experiments [12]. However, we have argued that it is unlikely to be a good system for supporting a protocell, as it is first-order, and catalysts from inside a cell are likely to escape and initiate the reaction in the environment, thereby destroying the necessary difference between the inside and outside. Another candidate for an autocatalytic reaction is the reverse TCA cycle, which appeared very early in evolutionary history. Although some of the reaction steps have been observed to occur without enzymes [13], there is still no demonstration of a complete cycle in a protocell without enzymes. In contrast, there are several demonstrations that non-enzymatic RNA replication can occur inside protocells if driven by a supply of activated nucleotides [14,15,16]. We have shown that as non-enzymatic replication is autocatalytic, this can itself constitute a metabolism, in which case oligomer templating leads to the origin of both metabolism and replication [10].

However, we will leave aside these questions of metabolism versus replication because the focus of the current paper is on the growth and division of the lipid membrane and not on the nature of the metabolic reaction. As we assume that lipids are synthesized chemically outside the cell, the membrane behavior is largely independent of the metabolism. We, therefore, use the simplest possible model of an IO-stable autocatalytic reaction in this paper, without specifying the chemistry it represents.

We now consider processes that allow vesicle growth and division. For a solution of amphiphilic lipids, there is a critical aggregation concentration (or critical vesicle concentration) above which membranes spontaneously form [17,18,19]. We denote this as $C^{*}$. If the total concentration is initially greater than $C^{*}$, we expect vesicles to form until the remaining concentration of dissolved molecules falls to $C^{*}$. At this point, the membranes are in equilibrium with the solution, with equal rates of molecules entering and leaving the membranes. If additional lipids are added to a solution containing vesicles, the existing vesicles increase in membrane area. This can result in elongated, non-spherical vesicles, or sometimes in division into smaller daughter vesicles [20,21,22,23].

Whether division occurs when the membrane area increases depends on the elasticity and curvature energies of the membrane, which we consider carefully in this paper. The area difference elasticity model has been used to calculate the minimum energy shapes of a vesicle and to determine when it is energetically favorable for a large vesicle to divide [24,25,26,27,28]. This includes terms for the curvature energy and for the elastic energy, which depends on the small difference in lipid areas $∆A_{lip}$ between the inner and outer leaflets of the bilayer. Division usually increases the curvature energy because smaller vesicles have a smaller radius of curvature. However, division can decrease the elastic energy in cases where a large $∆A_{lip}$ has built up in the parent cell. Lipids from the solution will enter the outer leaflet of a growing vesicle and gradually flip across to the inner leaflet. The area difference $∆A_{lip}$ is sensitive to the rate of lipid addition and the rate of flipping between leaflets. We show that vesicle division occurs most easily when the flipping is slow. The ratio of sizes of the two daughter vesicles is also sensitive to the flipping rate.

In this paper, we assume that vesicle division occurs when the change in energy occurring during vesicle division is negative. From a theoretical point of view, the energy change depends on the area difference; therefore, it is necessary to use a model that keeps track of the areas of both the inner and outer leaflets. There is also a lot of experimental evidence that shows that the difference between the leaflets is important. For example, the area difference is an essential parameter to interpret observed changes in vesicle shape [9,29]. The addition of lipids does not always cause division. One study observed that unilamellar vesicles grow to elongated shapes without division but multilamellar vesicles form tubular projections that subsequently divide [30]. Other studies showed that the osmotic deswelling of vesicles (which also increases the surface area-to-volume ratio, as with lipid addition) only leads to division if it is accompanied by a pH increase inside the vesicle [31,32], and that either of these factors alone is not sufficient. It appears that raising the internal pH increases the degree of ionization of the fatty acids, and that ionized fatty acids pass from the inner leaflet to the internal solution, which creates a larger area difference and favors division. Vesicle division can also be induced by a temperature increase, which causes membrane area expansion [33,34]. This can only be explained if the thermal expansivity of the outer leaflet is higher than that of the inner leaflet, so that the area difference increases after the temperature increase. In a similar way, vesicles composed of mixtures of cylindrical and inverse cone-shaped lipids can divide after osmotic deswelling if there is an excess of the inverse cone-shaped lipids on the inner leaflet [35,36]. All of these experiments point to the need for taking account of the asymmetry between the leaflets.

Another important point is that when membranes are under tension (as they will be if the vesicles are swollen by osmotic pressure), they have an increased tendency to absorb lipids. This is presumably because lipid addition decreases the elastic energy of a stretched membrane. It was shown [37] that vesicles swollen by an osmotic pressure can increase the lipid area while relaxed vesicles in the same solution decrease in area. This implies that active protocells can outcompete empty vesicles when there is a limited supply of lipids. Other studies have confirmed that membrane tension promotes the addition of new lipids [38,39]. In the model used here, a membrane under tension can gain lipids when the solution concentration is $C^{*}$, whereas a relaxed vesicle has a fixed area. If C is slightly below $C^{*}$, a membrane under tension can gain lipids while a relaxed vesicle shrinks, as in [37].

If osmotic pressure exists in a vesicle but the addition of extra lipids is too slow to keep up with the volume increase, then the membrane is under elastic tension. Lipid membranes can be stretched only to a limited degree and will burst if the tension is too high. Repeated bursting and re-sealing of vesicles has been observed, which gradually releases the internal pressure [40,41].

The aim of the current paper is to produce a simple computational model of a protocell that considers how membrane growth and division are related to osmotically driven volume growth. We want to determine when repeated cycles of growth and division can occur while the internal metabolism is sustained. Previous protocell models [42,43,44] have emphasized the need for synchronization between the reproduction of the cell’s contents and the membrane. There have also been more detailed stochastic simulations of protocells incorporating metabolic networks and membrane growth [45,46,47,48,49]. Our approach is to keep the model simple enough to be described by a relatively small number of differential equations. We focus particularly on the physics of membrane growth and division, as we expect these things to be generally applicable regardless of the nature of the autocatalytic system that is driving cell growth.

## 2. Methods

### 2.1. Fixed-Volume Compartment

Before considering cell growth, we need a model for an autocatalytic reaction in a compartment of a fixed volume. We use a model from our previous work [10], which is the simplest example that maintains a stable difference between the inside and outside of the cell. There are three kinds of molecules in the compartment, the food, catalyst, and waste, with respective concentrations of $C_{1}$, $C_{2}$, and $C_{W}$. In the environment, there is a fixed concentration $E_{1}$ of the food, and the catalyst and waste have concentrations of zero. Each catalyst is formed from two food molecules. The rate of the uncatalyzed reaction $2C_{1}\to C_{2}$ is assumed to be negligible, and the catalyst is formed by an autocatalytic process, which can be written as $2C_{1}+2C_{2}\to 3C_{2}$. This mechanism requires two molecules of the catalyst to make a third. The two catalyst molecules are not consumed by the reaction but the two food molecules are converted into a third catalyst. The rate of this reaction is $kC_{2}^{2}C_{1}^{2}$, i.e., it is second-order in the catalyst concentration. In addition, the catalyst decays into two molecules of waste, $C_{2}\to 2C_{W}$, at a rate of $wC_{2}$. The food molecule can enter and exit the cell with permeability $\mu_{1}$ and the waste molecule can exit the cell with permeability $\mu_{W}$, while the membrane is impermeable to the catalyst. This gives the following differential equations.

(1a) $$\frac{dC_{1}}{dt}=\mu_{1}(E_{1}-C_{1})-2kC_{1}^{2}C_{2}^{2}$$

(1b) $$\frac{dC_{2}}{dt}=kC_{1}^{2}C_{2}^{2}-wC_{2},$$

(1c) $$\frac{dC_{W}}{dt}=2wC_{2}-\mu_{W}C_{W},$$

Appendix A.1 gives the solution to Equations (1a)–(1c). There is a critical reaction rate $k_{c}$, such that for $k>k_{c}$, an active state is maintained inside the cell, with the non-zero catalyst concentration $C_{2}$, while for $k<k_{c}$, the reaction collapses inside the cell and the internal concentrations become the same as the external concentrations: $C_{1}=E_{1},\;C_{2}=0,\;C_{W}=0$. The behavior of the system (1a)–(1c) is generic for IO-stable systems. Many other examples have been discussed in [10]. In the current paper, we use the simplest generic model for autocatalysis. The main object of this paper is to extend the model of a fixed-volume compartment to a growing protocell.

### 2.2. Vesicle Area, Volume, and Shape

We consider a vesicle with different numbers of molecules $N^{+}$ and $N^{-}$ in the outer and inner leaflets of the bilayer. If a is the preferred area per lipid molecule, the natural areas of the leaflets when the membrane is relaxed are $A_{lip}^{+}=N^{+}a$ and $A_{lip}^{-}=N^{-}a$, and the natural area measured at the midpoint of the membrane is $A_{lip}=(A_{lip}^{+}+A_{lip}^{-})/2$. The actual area of the vesicle is a function of its shape and volume. The minimum possible surface area of a vesicle with volume V is the area of a sphere with that volume: $A_{sph}\left(V\right)={\left(36\pi \right)}^{1/3}V^{2/3}$. The actual area of the vesicle is $A=max(A_{sph}(V),A_{lip})$. If $A_{sph}(V)>A_{lip}$, the vesicle is a swollen sphere, and the membrane is under tension (the area per lipid is greater than the preferred area a). If $A_{lip}>A_{sph}(V)$, the membrane is relaxed, and the vesicle has an elongated, non-spherical shape.

The radius of the curvature of the membrane, R, is defined at the midpoint of the bilayer. The length of a lipid molecule is defined as 2d, so the total membrane thickness is 4d. Following [24], the actual areas of the outer and inner leaflets are determined at radii R+d and R−d, which are the midpoints of the two leaflets. These areas depend on the shape. We can write $A_{shape}^{+}=A+\frac{∆A_{shape}}{2}$ and $A_{shape}^{-}=A-\frac{∆A_{shape}}{2}$, where the shape-dependent area difference $∆A_{shape}$ is calculated in Appendix A.4. When the vesicle is non-spherical, we describe its shape as a capsule consisting of two hemispheres and a cylinder. If V and A are given, the shape of the capsule is determined (see Appendix A.3). There are many more complex shapes possible for a non-spherical vesicle [24,25,26]. We have chosen the capsule shape because the areas and energies can be determined rapidly without complex integrals. We need to know the shape area difference $∆A_{shape}$ and the membrane energy continuously at each point in time when we solve the differential equations. Therefore, we require a simple, rapid method of determining the changing shape.

The lipid area difference is determined by the numbers of lipids in the two leaflets: $∆A_{lip}=A_{lip}^{+}-A_{lip}^{-}$. This can change due to the exchange of lipids between the membrane and solution, and the flipping of lipids between the leaflets. If the shape difference were fixed, then $∆A_{lip}$ would tend to $∆A_{shape}$, which would minimize the elastic energy of the membrane. However, these two area differences are not always equal. According to the area difference energy model, the elastic energy of a vesicle depends on the difference between $∆A_{lip}$ and $∆A_{shape}$. Below, we will use this model to determine the conditions under which it is energetically favorable for a vesicle to divide.

### 2.3. Model for a Growing Protocell

We need to modify Equations (1a)–(1c) to account for changes in vesicle size. We define a reference vesicle of radius $R_{0}$, with an area and volume $A_{0}=4\pi R_{0}^{2}$ and $V_{0}=4\pi R_{0}^{3}/3$. The volumes and areas are measured as multiples of this. The differential equations for the growing vesicle are:

(2a) $$\frac{d}{dt}\left(\frac{V}{V_{0}}\right)=\lambda \left(\frac{A}{A_{0}}\right)∆C,$$

(2b) $$\frac{d}{dt}\left(\frac{A_{lip}^{+}}{A_{0}}\right)=r_{mem}\left(\frac{E_{lip}}{C^{*}}\frac{A_{shape}^{+}}{A_{0}}-\frac{A_{lip}^{+}}{A_{0}}\right)-r_{flip}\left(\frac{A_{shape}^{-}}{A}\frac{A_{lip}^{+}}{A_{0}}-\frac{A_{shape}^{+}}{A}\frac{A_{lip}^{-}}{A_{0}}\right)$$

(2c) $$\frac{d}{dt}\left(\frac{A_{lip}^{-}}{A_{0}}\right)=r_{mem}\left(\frac{C_{lip}}{C^{*}}\frac{A_{shape}^{-}}{A_{0}}-\frac{A_{lip}^{-}}{A_{0}}\right)+r_{flip}\left(\frac{A_{shape}^{-}}{A}\frac{A_{lip}^{+}}{A_{0}}-\frac{A_{shape}^{+}}{A}\frac{A_{lip}^{-}}{A_{0}}\right)$$

(2d) $$\frac{dC_{1}}{dt}=\mu_{1}s\left(E_{1}-C_{1}\right)-2kC_{1}^{2}C_{2}^{2}-C_{1}\lambda s∆C$$

(2e) $$\frac{dC_{2}}{dt}=kC_{1}^{2}C_{2}^{2}-wC_{2}-C_{2}\lambda s∆C$$

(2f) $$\frac{dC_{W}}{dt}=2wC_{2}-\mu_{W}sC_{W}-C_{W}\lambda s∆C$$

(2g) $$\frac{dC_{lip}}{dt}=-r_{mem}Q\left(\frac{V_{0}}{V}\right)\left(\frac{C_{lip}}{C^{*}}\frac{A_{shape}^{-}}{A_{0}}-\frac{A_{lip}^{-}}{A_{0}}\right)-C_{lip}\lambda s∆C$$

Equation (2a) assumes that the rate of volume growth due to osmosis is proportional to the surface area and the difference in concentration $∆C=C_{1}+C_{2}+C_{W}+C_{lip}-E_{1}-E_{lip}$. The external food and lipid concentrations are fixed at $E_{1}$ and $E_{lip}$. The internal concentrations are variable. Here, λ is the rate constant for volume growth by osmosis.

Equations (2b) and (2c) for the membrane areas depend on the rates $r_{mem}$ for the exchange of lipids between the membrane and the solution and $r_{flip}$ for the flipping of lipids between the leaflets. If we assume that the flipping is always very fast, it would be possible to reduce these two equations to a single equation that ignores the area difference between the leaflets. However, the asymmetry between the leaflets seems to be an important feature that influences division (as discussed in the Introduction). In our view, treating the leaflets separately is an important novel feature of this model. If the outside concentration is maintained at $E_{lip}>C^{*}$ due to the continued input of new lipids to the environment, then the membrane will be relaxed, with $A_{shape}^{+}=A_{lip}^{+}$, and the first term in Equation (2b) gives an increase in membrane area at a rate proportional to the excess lipid concentration ${(E}_{lip}/C^{*}-1)$. If $E_{lip}<C^{*}$, a relaxed membrane will shrink. However, if the membrane is under tension (which is the case when the there is a positive osmotic pressure), then $A_{shape}^{+}>A_{lip}^{+}$, so it is possible for a swollen vesicle to increase in lipid area even when $E_{lip}<C^{*}$.

The second term in (2b) describes the flipping of lipids between leaflets. For a flat membrane, $A_{shape}^{+}=A_{shape}^{-}$; therefore, the steady state has $A_{lip}^{+}=A_{lip}^{-}$. However, for a curved membrane the shape areas are not equal, and the steady state has $\frac{A_{lip}^{+}}{A_{shape}^{+}}=\frac{A_{lip}^{-}}{A_{shape}^{-}}$, which means that the densities of lipids in the two leaflets are equal.

The permeability term $\mu_{1}\left(E_{1}-C_{1}\right)$ from (1a) is multiplied by a factor s in (2d). This is the dimensionless surface area-to-volume ratio: $s=\frac{(A/A_{0})}{(V/V_{0})}$. The factor of s arises because the flux of the molecules through the membrane is proportional to the area, although the change in concentration is inversely proportional to the volume. The internal lipid concentration $C_{lip}$ also changes as a result of the exchange of lipids with the inner leaflet. If the inner area $A_{lip}^{-}$ changes by δA, then the number of molecules exchanged is δA/a and the change in molar concentration of the lipids is ${\delta C}_{lip}=-\delta A/(an_{Av}V)$, where $n_{Av}$ is Avogadro’s number and V is the volume in litres. Expressing areas and volumes relative to the standard vesicle, $\delta C_{lip}=-Q\frac{V_{0}}{V}\frac{\delta A}{A_{0}}$, where the constant $Q=\frac{A_{0}}{an_{Av}V_{0}}$, which appears in the first term of (2g).

The concentrations also change due to dilution when the volume increases. This gives a term $-\frac{C}{V}\frac{dV}{dt}$ in each of the equations for the concentrations ((2d)–(2g)). Using Equation (2a), this can be written as −Cλs∆C.

Through the numerical solution of (2a)–(2g), we can follow the changes in the internal concentrations of the reagents at the same time as the volume and area are changing. When using these equations, it should be remembered that $A_{lip}^{+}$ and $A_{lip}^{-}$ are independent variables, although the shape areas $A_{shape}^{+}=A+\frac{∆A_{shape}}{2}$ and $A_{shape}^{-}=A-\frac{∆A_{shape}}{2}$ are determined by assuming a capsule shape, as in Appendix A.3 and Appendix A.4.

### 2.4. Vesicle Division and Bursting

A relaxed vesicle has an actual area of $A_{lip}$. The radius of a sphere that has this area is $R_{sph}={\left(\frac{A_{lip}}{4\pi }\right)}^{1/2}$. The volume of a sphere with this area is $V_{sph}=\frac{4\pi }{3}R_{sph}^{3}=\frac{1}{6\sqrt{\pi }}A_{lip}^{3/2}$. The reduced volume is defined as $v=V/V_{sph}$. When v=1, the vesicle is a relaxed sphere. When v<1, the vesicle is a capsule, which becomes longer and thinner as v decreases. When v>1, the vesicle is a swollen sphere. In terms of the standard vesicle volume and area, we can write $\frac{V_{sph}}{V_{0}}={\left(\frac{A_{lip}}{A_{0}}\right)}^{3/2}$, so $v=\frac{(V/V_{0})}{{\left(A_{lip}/A_{0}\right)}^{3/2\;}}$. For a swollen sphere, $\frac{V}{V_{0}}=\frac{V_{sph}}{V_{0}}={\left(\frac{A_{sph}}{A_{0}}\right)}^{3/2}$ and $v={\left(\frac{A_{sph}}{A_{lip}}\right)}^{3/2}$. We suppose that the membrane can only be stretched by a limited factor and that bursting occurs when $\frac{A}{A_{lip}}=1.1$, or when $v=1.1^{3/2}\approx 1.15$. Bursting forms a temporary pore through which some of the vesicle contents are released, although it does not destroy the vesicle. We suppose that 10% of the internal volume is lost on bursting, after which the membrane recloses, as was observed in experiments with pulsatile vesicles [40]. We suppose that no lipids are lost from the membrane during bursting, and that no external solution enters through the pore. Therefore, the lipid area remains constant and the internal concentrations are unchanged by bursting.

**Figure 1:**
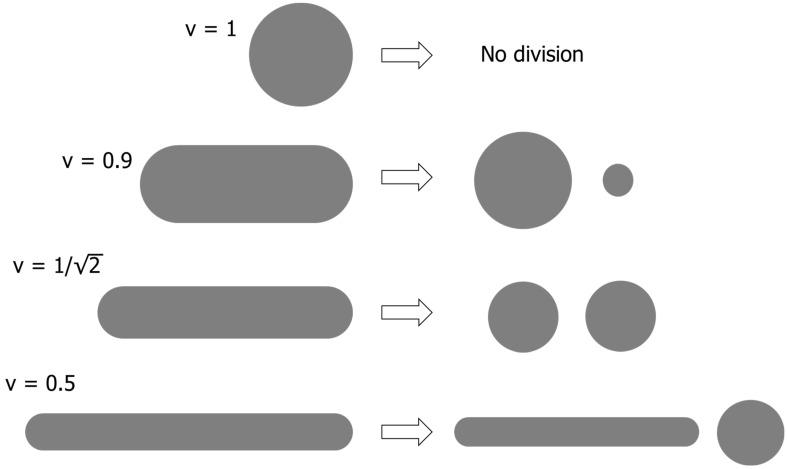
Possible means of cell division that conserve both the volume and area.

We now consider vesicle division. We assume that the combined areas and volumes of the two daughter cells after division are equal to those of the parent. A spherical cell with a reduced volume v=1 cannot divide because the parental area is insufficient to enclose two smaller cells. When $\frac{1}{\sqrt{2}}<v<1$, it is possible for the elongated parent cell to divide into two daughter cells that are spheres of unequal size (as shown in Figure 1). When $v=\frac{1}{\sqrt{2}}$, the parent can form two equal-sized spheres. When $v<\frac{1}{\sqrt{2}}$, it is possible to form one sphere and one elongated daughter cell.

When we follow the growth of a vesicle with Equations (2a)–(2g), we know the reduced volume v at any point. The simplest rule for division, which we call the “equal division rule”, is that the cell divides into two equal-sized spheres whenever v reaches $\frac{1}{\sqrt{2}}$. We also use an “energy decrease rule”, which takes account of the energy of the membranes. Using the area difference energy model, the combined curvature and elasticity energy can be calculated as a function of the volume and area parameters (as described in Appendix A.4). We suppose that there is an attempted division rate $r_{div}$, so that in each time step δt there is a probability $r_{div}\delta t$ of an attempted division. When this occurs, we calculate the energy of the parent vesicle before division, $E_{par}$, and the energies $E_{1}$ and $E_{2}$ of the daughter vesicles. The change in energy is $∆E=E_{1}+E_{2}-E_{par}$. We allow the attempted division if ∆E≤0, otherwise the attempt is unsuccessful, and the parental vesicle remains unchanged.

**Figure 2:**
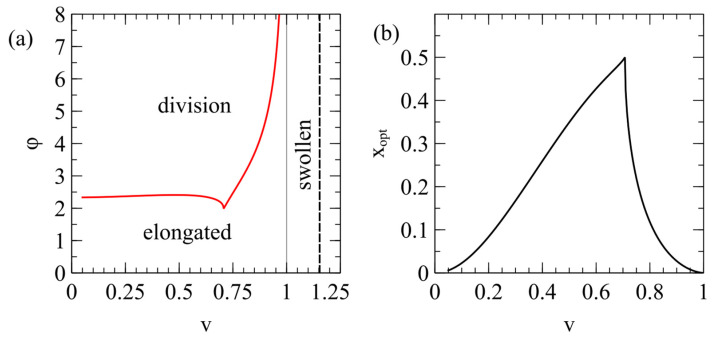
(**a**) Phase diagram for the area difference energy model. When v=1, the vesicle is a relaxed sphere. For 1<v<1.15, the vesicle is a swollen sphere. Bursting occurs at $v=1.1^{3/2}\approx 1.15$. For v<1, the vesicle is elongated (modelled as a capsule shape). The red line is $\phi_{min}(v)$, the point at which ∆E=0. Division is energetically favorable for $\phi >\phi_{min}(v)$. (**b**) The optimal ratio of sizes of the daughter vesicles $x_{opt}$, which minimizes their elastic energy.

Here, ∆E is a function of two parameters—the reduced volume v and the area difference parameter $\phi =∆A_{lip}/∆A_{shape}$. For a given value of v, there is a minimum value $\phi_{min}(v)$, such that ∆E≤0 only when $\phi \geq \phi_{min}(v)$ (see Appendix A.5). This is shown in Figure 2a. The cusp of this curve occurs at $v=1/\sqrt{2}$. This is the point at which division into two equal spheres is energetically favorable. The right branch of the curve for $\frac{1}{\sqrt{2}}\leq v<1$ is the point at which it becomes favorable to divide into two spheres of unequal size, and the left branch of the curve for $v<\frac{1}{\sqrt{2}}$ is the point at which it becomes favorable to divide into one sphere and one capsule. Figure 2b shows the optimal ratio of volume $x=x_{opt}$ of the two daughters for which the elastic energy is minimized. This is 0.5 for $v=\frac{1}{\sqrt{2}}$, and otherwise is less than 0.5 (see Appendix A.5).

### 2.5. Parameter Values and Simulation Methods

It is convenient to measure the vesicle size relative to a reference vesicle with $R_{0}=1\;\mu m$. The sizes of experimental vesicles vary considerably from >10 μm for giant unilamellar vesicles [26,29,40,41] to around radius 50 nm for fatty acid vesicles [30,37], so this is in the right range. However, the behavior of the model does not depend on the choice of $R_{0}$. We choose d=1 nm, so the membrane thickness is 4 nm. The area per lipid is $a=2\times 10^{-19}m^{2}$. The critical aggregation concentration is $C^{*}=0.01\;M$. These parameters are approximately correct for fatty acid membranes [17,18], although for modern phospholipids, d is somewhat larger and $C^{*}$ is significantly smaller. The parameter $Q=\frac{A_{0}}{an_{Av}V_{0}}$ is then 0.0249. The external lipid concentration $E_{lip}$ may be slightly higher or lower than $C^{*}$, and varies in the examples below. The external food concentration is $E_{1}=1.0\;M$, which is much higher than $C^{*}$, so the osmotic pressure is mostly controlled by the reactants $C_{1},\;C_{2},\;{and\;C}_{W}$ and not by the lipid.

The standard parameter set for the reaction rates and permeabilities is: $k=1.0,\;w=0.01,\;\mu_{1}=0.01,\;\mu_{W}=0.015$. We do not give units here because the time scale is arbitrary for this idealized reaction. The volume expansion rate is λ=1, and the rates for lipid dynamics are expressed as $r_{mem}=r_{flip}=0.1$. These parameters allow successful growth in volume and area without bursting. When parameters are varied relative to this standard set, we will state so below.

We use the fourth-order Runge–Kutta method for the ODEs (2a)–(2g), with a time step of δt=0.001. Initially, the vesicle size is set to the reference vesicle size, $V=V_{0},\;A_{lip}=A_{0}$; the internal lipid concentration is $C_{lip}=C^{*}$; and the concentrations $C_{1},\;C_{2},\;{and\;C}_{W}$ are set to the steady-state concentrations for the fixed-volume compartment discussed in Section 2.1 and Appendix A.1.

## 3. Results

### 3.1. Protocell Growth Without Division

**Figure 3:**
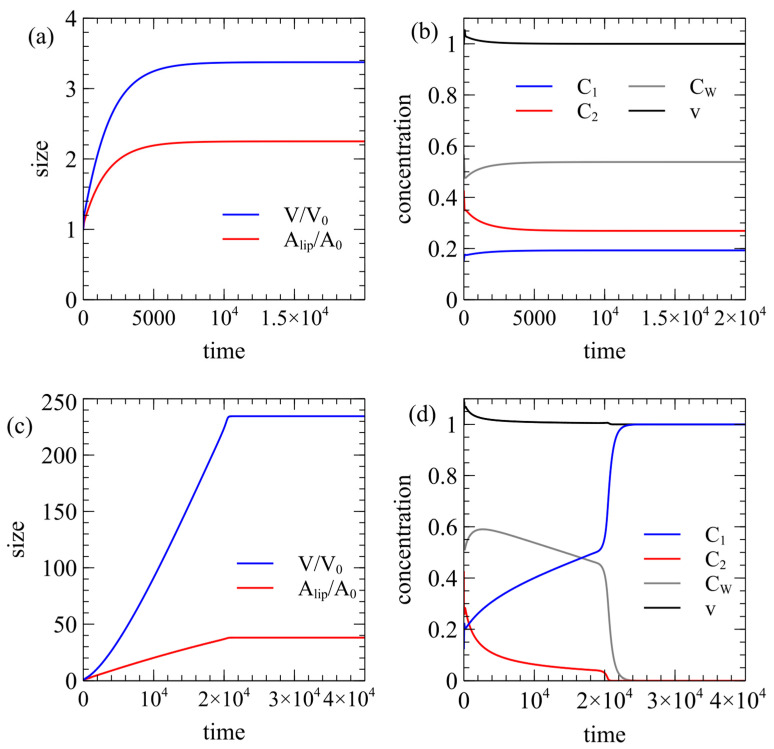
Growth of a lipid vesicle with the external lipid concentration $E_{lip}=C^{*}$. (**a**,**b**) An example leading to a homeostatic state where the internal reactions are maintained but there is no further growth. Standard rate parameters. (**c**,**d**) An example showing growth to a very large size, after which the internal reaction collapses and growth stops. Standard parameters except $\mu_{W}=0.1$.

Figure 3 considers growth driven by the autocatalytic reaction when division does not occur. Figure 3a uses the standard rate parameters (Section 2.5). The volume increases initially because the reaction creates a positive ∆C. The external lipid concentration is $E_{lip}=C^{*}$, so a relaxed membrane cannot grow. However, the membrane is under tension, and it is able to grow because $A_{shape}^{+}>A_{lip}^{+}$. In Figure 3a, the vesicle stops growing when it reaches a steady state of homeostasis in which the internal reaction is maintained but there is no further change in volume. Figure 3b shows that the internal concentrations $C_{1},\;C_{2}$, and $C_{W}$ all remain stable in the steady state.

The homeostatic state does not always exist in our model. In Figure 3c, the permeability of the waste is changed to $\mu_{W}=0.01$, whereas it was 0.015 in Figure 3a. In this case, both the volume and area become extremely large but the internal concentrations do not reach a stable state, and eventually the autocatalytic reaction collapses, as shown in Figure 3d. After the collapse, the internal concentrations become the same as the external concentrations. The cell dies because it becomes too big to sustain itself. When a cell grows, the surface area-to-volume ratio decreases. The reaction cannot be maintained if the cell is too large because the food supply rate is proportional to the area and the food consumption rate is proportional to the volume. In Appendix A.2, we give an analytical solution determining when the homeostatic state occurs.

In both cases in Figure 3, the external lipid concentration is $E_{lip}=C^{*}$, so the cell only grows because the internal reaction creates an osmotic pressure that places the membrane under tension. If an inactive cell is initiated with internal concentrations equal to external concentrations, then there is no osmotic pressure and no tension in the membrane. In this case, the inactive cell does not grow in either area or volume. This is consistent with the experiments in [37], which found that vesicles containing a positive osmotic pressure increase in lipid area relative to those with no osmotic pressure. This is relevant for competition between active cells and empty vesicles. However, as the active cells are growing with v≥1, they never reach the region where division is possible in Figure 2a. Thus, this theory predicts that when $E_{lip}=C^{*}$, an active cell can grow but cannot divide.

### 3.2. Protocell Growth with Division

**Figure 4:**
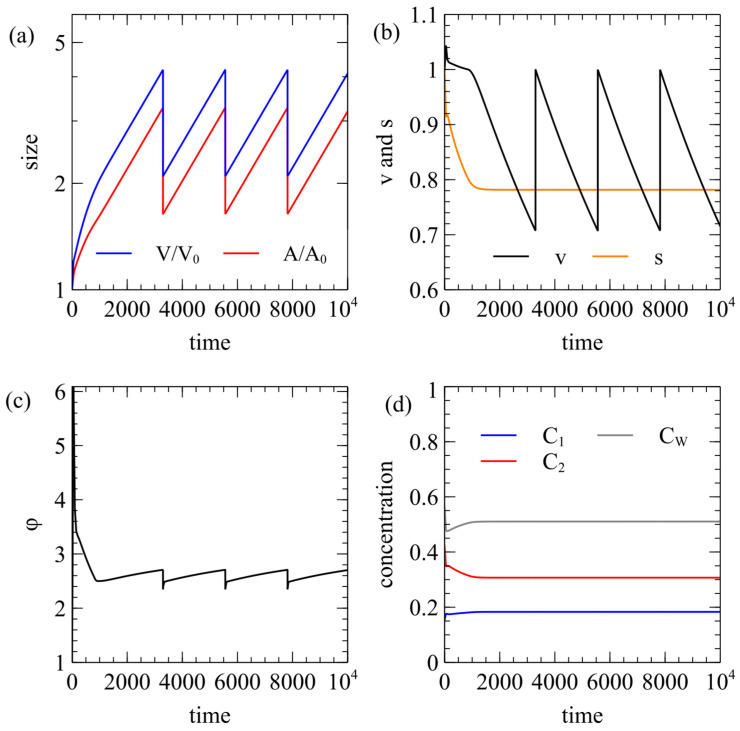
Time course of a cell using the equal division rule. Standard rate parameters with $E_{lip}=1.01C^{*}$. (**a**) Volume and area quickly reach a state where they grow in proportion to one another and halve at each division point. (**b**) Surface area-to-volume ratio s reaches a constant value. Reduced volume *v* descends to $1/\sqrt{2}$ at each division point. (**c**) Area difference parameter ϕ is relatively stable over cycles. (**d**) Internal reactant concentrations reach a steady state.

For a cell to divide it must become elongated, so it has to be possible for the area to increase when the membrane is relaxed. Therefore, the external lipid concentration must be greater than $C^{*}.$ In this section, we consider examples where $E_{lip}=1.01C^{*}$, and we show that even this slight increase above $C^{*}$ is sufficient to allow division. In Figure 4, beginning with a spherical vesicle, there is a short period of growth as a swollen sphere, after which it becomes a capsule shape in which the volume and area grow exponentially at the same rate (V and A are parallel on a log scale in Figure 4a). This means that the surface area-to-volume ratio s becomes constant while v decreases (Figure 4b).

We use the equal division rule, in which division occurs when $v=\frac{1}{\sqrt{2}}$. At this point, the volume V and the membrane areas $A_{lip}^{+}$ and $A_{lip}^{-}$ are halved. This creates two equal-sized spheres, and the program continues to follow one of these after division. Since division forms spheres, v is reset to 1 during division but it then decreases again until another division point is reached. Figure 4c shows the area difference parameter ϕ, (although the equal division rule does not depend on ϕ). Figure 4d shows that the internal concentrations become constant. As the surface area-to-volume ratio is constant, the food supply is balanced by consumption at constant concentrations as the cell grows.

**Figure 5:**
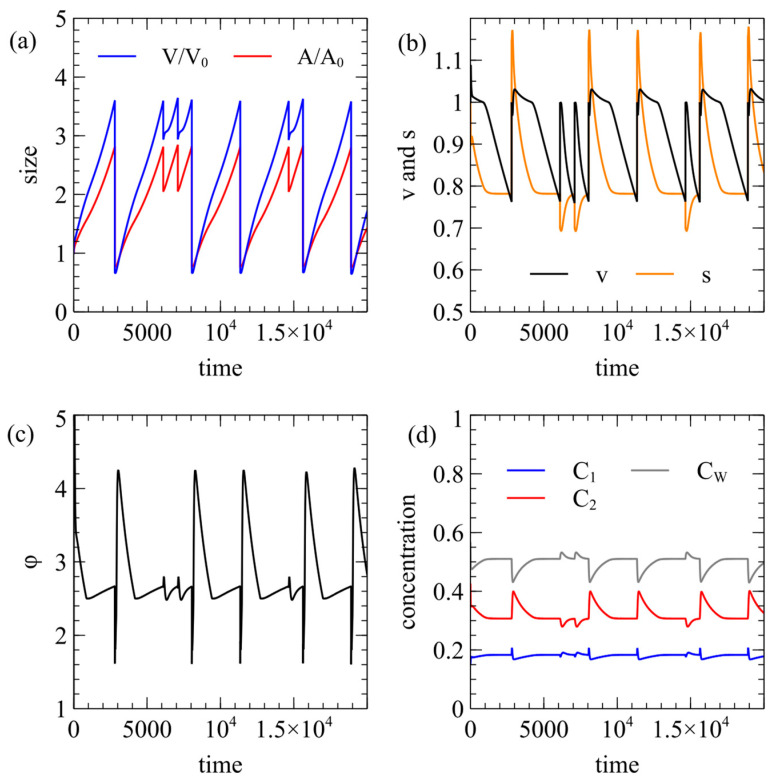
Time course of a cell using the energy decrease rule. The parameters are as in Figure 4. (**a**) At each division point, we randomly follow either the larger or smaller of the two daughter vesicles, which is visible by either small or large drops in volume. (**b**–**d**) The other vesicle properties fluctuate somewhat after each division but return to the same typical values before the next division occurs.

Figure 5 shows an example with the same parameters as Figure 4 using the energy decrease rule instead of equal division. In this case, when division occurs, the two daughter cells are different sizes, and we follow either the larger or the smaller daughter at random. When the smaller cell is followed, it takes longer to grow back to the next division point than when the larger cell is followed. However, the sizes of the cells at the next division point are approximately the same in both cases. The attempted division rate is kept high, $r_{div}=10$, so successful divisions occur very soon after the vesicle crosses the boundary line in Figure 2a. The values of v and ϕ at which division occurs are almost the same each time. Figure 5d shows that the internal concentrations are disrupted after each division event but they trend fairly rapidly towards stable values prior to the next division event.

**Figure 6:**
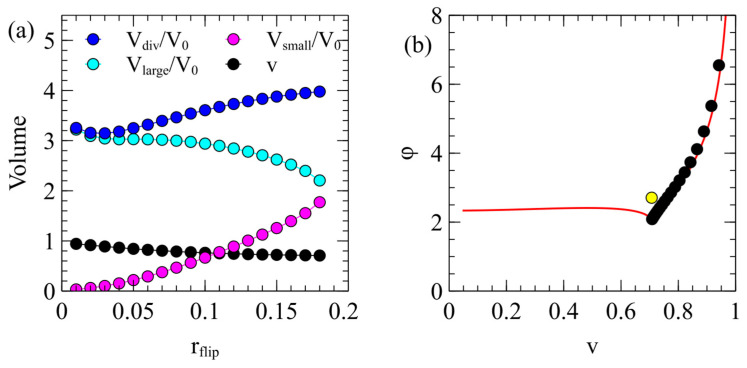
(**a**) Mean properties of cells at the division point as a function of $r_{flip}$, with $r_{mem}=0.1$. The other parameters are standard. (**b**) Mean values of (v,ϕ) at which divisions occur. Black points are for the energy decrease rule, with $r_{flip}$ varying from 0.01 to 0.18. The yellow point is for the equal division rule, with $r_{flip}=r_{mem}=0.1$, as in Figure 4.

As ∆E depends on the area difference parameter ϕ, the cell division process is sensitive to the rate of flipping of the lipids between the two leaflets of the bilayer, $r_{flip}$. We ran several simulations with different values of $r_{flip}$, keeping all the other parameters the same as those in Figure 4 and Figure 5. We followed either the larger or the smaller daughter at random each time division occurred. The mean properties of the cells at the point of the division (Figure 6) were found by averaging them over many division events. $V_{large}$ and $V_{small}$ are the mean sizes of the larger and smaller daughter vesicles immediately after division. $V_{div}$ is the mean size of the parent vesicle at the point of division. For smaller values of $r_{flip}$, the division is very unequal, $V_{small}\ll V_{large}$, and these sizes become more even as the flipping rate increases. The parent vesicle size becomes slightly larger as $r_{flip}$ increases because it takes slightly longer for ϕ to reach the point where division becomes favorable. The reduced volume v of the parent vesicle at the division point is also shown in Figure 6a. This is close to 1 for the small $r_{flip}$ value and decreases to $\frac{1}{\sqrt{2}}$ as $r_{flip}$ increases.

Figure 6b shows the mean values of (v,ϕ) for dividing cells on the phase diagram. The mean division points are all very close to the boundary line $\phi_{min}(v)$, meaning that division occurs almost as soon as it becomes energetically favorable. The division ratio x is always close to $x_{opt}$ because ∆E is negative only when x is close to $x_{opt}.$ Therefore, there is a well-defined division ratio for each value of $r_{flip}$. The smallest flipping rate considered is $r_{flip}=0.01$, which corresponds to the point on Figure 6b with the highest ϕ and the largest v. The points move down the boundary line as $r_{flip}$ increases and reach the cusp at $r_{flip}=0.18$. If $r_{flip}>0.18$, the trajectory of the vesicle on the phase diagram never crosses the boundary line, so division never occurs when $r_{flip}$ is too large.

The division point for the equal division rule is also shown as a yellow point in Figure 6b. This is well inside the boundary line. This means that the equal division rule is not forbidden by energetic considerations but division is unlikely to occur in this way, because if the attempted division rate $r_{div}$ is high, division is more likely to occur as soon as the trajectory crosses the boundary line. The point at which the boundary is crossed will not, in general, be at $v=\frac{1}{\sqrt{2}}$, so division will usually produce daughters of unequal sizes.

### 3.3. Examples Where Division Is Difficult or Not Sustained

**Figure 7:**
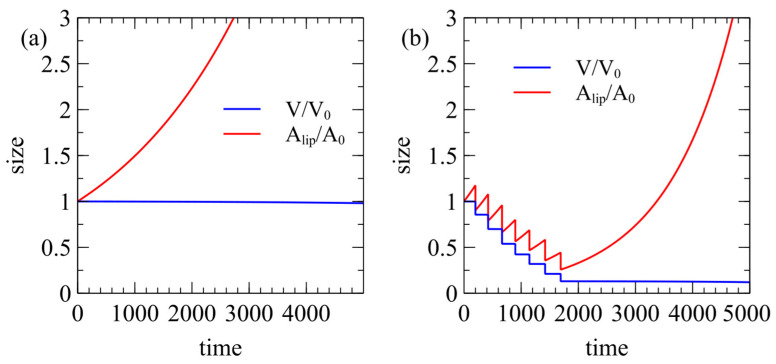
Inactive cells with no internal catalytic reaction may increase in membrane area if $E_{lip}>C^{*}$ but do not increase in volume. (**a**) When $E_{lip}=1.01C^{*}$, the vesicle increases in area at a fixed volume and never divides. (**b**) When $E_{lip}=1.02C^{*}$, the vesicle divides a few times, after which it increases in area without further division.

Figure 7 shows the behavior of inactive vesicles in which there is no catalytic reaction. These simulations were initiated with the internal food concentration equal to that outside, and with no catalyst. There is no osmotic pressure to drive growth. In Figure 7a, $r_{flip}=r_{mem}=0.1$, $E_{lip}=1.01C^{*}$, and all other parameters are as in Figure 4 and Figure 5. The area increases exponentially but the volume stays fixed. The vesicle never reaches the boundary line for division, so it becomes very long and narrow without dividing. However, it is not true that an inactive cell can never divide. In Figure 7b, we increase the external lipid concentration to $E_{lip}=1.02C^{*}$, with the rate constants remaining the same. This leads to several division events but the volume gets progressively smaller each time, until it reaches a stage of continued area growth without division. Thus, inactive cells do not reach a stable cycle of growth and division in the way that active cells do.

**Figure 8:**
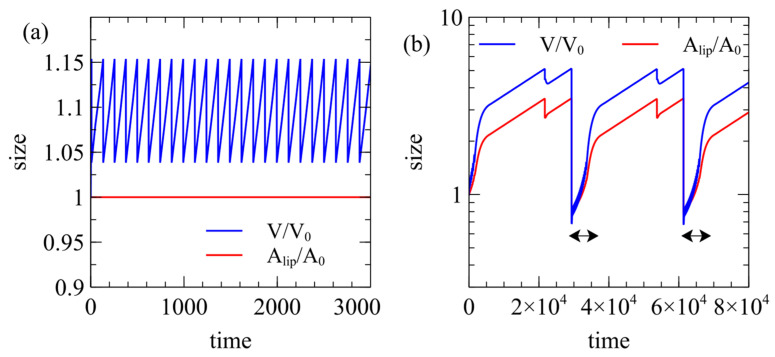
Cases where frequent bursting of vesicles occurs. (**a**) If $r_{mem}=r_{flip}=0.0$, there is no exchange of lipids with the solution, so the membrane area is fixed. The vesicle grows repeatedly to the bursting point but can never divide. (**b**) If $E_{lip}=1.01C^{*},r_{mem}=r_{flip}=0.01$, division produces small daughter cells that are subject to repeated rapid bursting events (periods indicated by arrows), although these eventually grow large enough for division.

If a vesicle is to reach the division point, it must also avoid bursting. In Figure 8a, we show an example of a vesicle with an active reaction inside where there is no exchange of lipids with the solution ($r_{mem}=r_{flip}=0$). The membrane area is fixed but the osmotic pressure drives the volume up to bursting point. The vesicle is trapped in repeated rapid cycles of bursting but it can never divide.

In Figure 8b, we consider a very slow lipid exchange, with $r_{mem}=r_{flip}=0.01$, which is ten times less than in Figure 4 and Figure 5. In this case, the cell grows to division much more slowly than in Figure 4 and Figure 5. Small daughter cells that are vulnerable to bursting form. Frequent rapid bursts occur in the periods indicated by the arrows in Figure 8b but the cell manages to grow slightly larger each time until it escapes the bursting cycle and slowly regrows to the division point.

## 4. Discussion

We have presented a model that unifies a minimal autocatalytic reaction network with a fatty acid vesicle incorporating lipids from the environment in order to explain the lifecycles of primitive protocells. The model shows that protocell division is not an inevitable consequence of membrane growth but instead emerges from a balance between lipid dynamics, osmotic stress, and membrane asymmetry. Our model emphasizes several requirements for a continued cycle of growth and division:

1. The cell must contain an active autocatalytic reaction. Cells with an active internal reaction reach a sustainable reproductive cycle with a constant mean size at the point of division (as in Figure 4, Figure 5 and Figure 6), whereas inactive vesicles with no internal reaction show membrane growth without volume growth. Inactive cells either divide into increasingly smaller vesicles or reach a state where division does not occur (as in Figure 7).
2. The rate parameters for the autocatalytic reaction and for the permeability of food and waste molecules must be such that the reaction is maintained inside the cell as it grows (as in Figure 3a,b), rather than collapsing when the cell becomes too large (as in Figure 3c,d).
3. The rate of entry of new lipids to the membrane must be sufficiently high relative to the rate of volume increase to avoid frequent bursting of the membrane and loss of cell contents (as in Figure 8).
4. Division only occurs if the area difference parameter ϕ becomes sufficiently large, which means that the division is sensitive to the flipping of lipids between outer and inner leaflets. It is necessary to have some degree of flipping for both leaflets to grow. However, if the flipping is too fast, ϕ remains close to 1, and vesicle division is not energetically favorable. Therefore, the reproductive cycle is facilitated by relatively slow flipping of lipids.
5. Division only occurs if the cell becomes sufficiently elongated (the reduced volume v becomes sufficiently small). This means that the external lipid concentration $E_{lip}$ must be above the critical aggregation concentration $C^{*}$, so that a relaxed membrane can increase in area. If the membrane is under tension due to an internal osmotic pressure, then the membrane can increase in area when $E_{lip}\leq C^{*}$. However, in this case, the cell remains spherical, so division is not possible.

The results presented here assume that $E_{lip}$ remains fixed. In order to maintain $E_{lip}$ higher than $C^{*}$, there must be a continued supply of new lipids to the environment, otherwise the concentration will reach an equilibrium at $C^{*}$. We intend to extend this model to consider a finite volume pond containing a population of many competing vesicles. In this case, the concentrations of lipid and food molecules in the pond will vary. It was shown experimentally [37] that active cells can outcompete inactive cells because active cells gain lipids under conditions where the inactive cells lose lipids. However, this mechanism only works when $E_{lip}\leq C^{*}$, i.e., competition for lipids only works in conditions where division cannot occur. We suggest that it is possible to achieve both competition for lipids and cell division if the supply of lipids comes in irregular batches, rather than a slow steady supply. If a batch of new lipids is added to the pond, there will be a short period where $E_{lip}>C^{*}$, so growth and division can occur. After this, $E_{lip}$ will fall quickly to $C^{*}$ or just less, in which case lipids will continue to be added to the stretched membranes of active cells and will be removed from inactive relaxed membranes. Over multiple batches of lipid addition, we expect active cells to outcompete inactive ones.

Another way to introduce fluctuations of lipid concentration is by wetting and drying the pond. If the pond partially dries out, the external concentration of food molecules (and any other solutes) will increase, creating a negative osmotic pressure, causing the vesicle to de-swell. De-swelling may sometimes induce division, although this will depend on the value of ϕ prior to de-swelling and on whether lipid flipping occurs during the period of de-swelling. It may also depend on other factors such as the internal pH [31,32] and shape of the lipid [35,36].

It is also known that de-swelling can cause division when the membrane is made of a mixture of lipids and phase separation occurs in the membrane. Division then happens along the boundary between the phases [50,51,52,53]. Our model applies either to a single kind of lipid or to a mixture of several lipids that does not separate. We have not considered the possibility of phase separation; however, if the division were dependent on phase separation, the composition of the two vesicles would be different, and these would not necessarily be able to divide a second time. Thus, phase separation does not seem ideal for achieving a sustainable cycle in protocells.

Another possibility that has been studied experimentally is that the new lipids that enter the membrane are synthesized by a mechanism that is catalyzed by the existing membrane [54,55,56]. This could be relevant for protocells but it does not seem an essential requirement for lipid synthesis to be autocatalytic in the simplest case. We have looked at the case where pre-formed lipids simply enter the membrane, which seems the simplest case to start with. The later evolutionary stages could involve switching to a mechanism of lipid synthesis on the membrane or inside the vesicle. The later stages could also involve the synthesis of different forms of lipids that improve the stability relative to simple fatty acid membranes [57].

Some of our results resemble those of Mavelli and Ruiz-Mirazo [45]. In their paper, only the equal division rule was used, whereas we have considered the role of the membrane curvature and elasticity energy in controlling division. The dependence of the lipid area growth on membrane tension is also a novel feature of our model, which seems to be important in real vesicles. Scheme 1 in [45], where an external lipid is synthesized from a precursor at a constant rate, is similar to the case with $E_{lip}>C^{*}$ considered here. However, in [45], there was no internal reaction with Scheme 1, so the cells divided and decreased in volume, similarly to the inactive cells in our Figure 7b. A state with reproducing cells that regrow to the same size was only found in [45] for Scheme 2 (membrane-catalyzed lipid synthesis) and Scheme 3 (internal lipid synthesis). In a subsequent paper [47], for the case of internal lipid synthesis, a stationary division regime was found in which the volume and area grow at the same rate, and in which the cell size during division remains constant over generations. This is also what happens in our model (Figure 4a and Figure 5a); however, we show here that reproducing cells of a steady size can exist even if the lipid supply is provided by the environment. Hence, the first protocells do not need to synthesize their own lipids, which is one fewer problem that needs to be dealt with by the earliest cells. It will obviously be an advantage if cells evolve to make their own lipids at a later state because this will make the cell less dependent on the environment. We also note an interesting model of autocatalytic formation of coacervate droplets [58], which has several similar properties to our model of lipid vesicles.

In summary, we have given a computational model of protocells powered by an internal autocatalytic reaction that drives cell growth and division. It has been kept intentionally simple so as to investigate the physics of membrane growth and division, allowing us to define the criteria necessary for division to occur. Cell division depends on a balance between lipid incorporation and lipid flipping. Our model hints at what needs to be achieved experimentally if a fully working protocell system is to be made in the laboratory. We plan to extend this model to study populations of protocells competing for resources under prebiotic conditions.

## Figures and Tables

**Figure A1:**
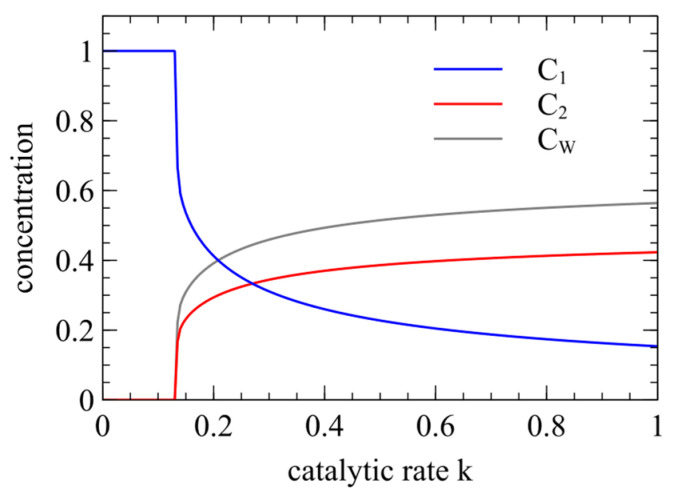
Steady-state concentrations for an autocatalytic reaction in a fixed-volume compartment. $E_{1}=1,\;w=0.01,\;\mu_{1}=0.01,\;\mu_{W}=0.015$.

**Figure A2:**
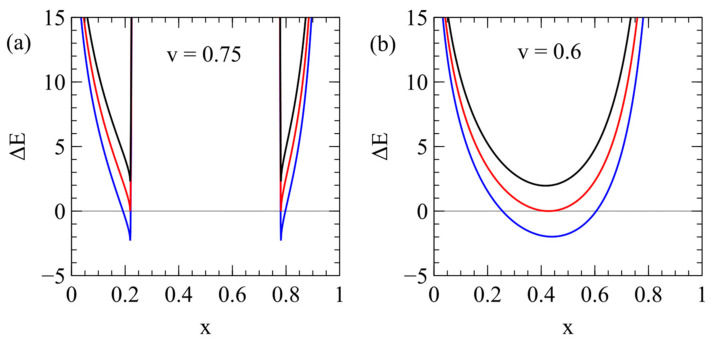
Changes in the curvature and elasticity energy ∆E as a function of the division ratio x for two different reduced volumes. In each case, the middle (red) curve is $\phi =\phi_{min}(v)$, the upper curve is $\phi =0.9\phi_{min}(v)$, and the lower (blue) curve is $\phi =1.1\phi_{min}(v)$: (**a**) v=0.75; (**b**) v=0.6.

## Data Availability

The program used for the modelling work in this paper is available at https://zenodo.org/records/15157714 (accessed on 1 April 2025).

## Appendix A. Mathematical Details

### Appendix A.1. Solution for the Fixed-Volume Compartment

Figure A1 shows the steady-state concentrations for Equations (1a)–(1c) as a function of the catalytic rate k. If k is less than the critical rate $k_{c}$, the reaction collapses inside the cell, and the internal concentrations are the same as the external concentrations: $C_{1}=E_{1},\;C_{2}=0,\;C_{W}=0$. For $k>k_{c}$, an active state is maintained inside the cell, with a non-zero catalyst concentration $C_{2}$.

From (1b), in the steady state, we have either an active state with $C_{2}=\frac{w}{kC_{1}^{2}}$ or an inactive state with $C_{2}=0$. In the active state, from (1c), we have $C_{W}=\frac{2w}{\mu_{W}}C_{2}=\frac{2w^{2}}{k\mu_{W}C_{1}^{2}}$. From (1a) and (1b), we have $2wC_{2}=\mu_{1}(E_{1}-C_{1})$, which can be written as

$$E_{1}=f\left(C_{1}\right)=C_{1}+\frac{2w^{2}}{k\mu_{1}C_{1}^{2}}$$

The required solution for $C_{1}$ is the smaller of the roots of $f\left(C_{1}\right)=E_{1}$. A solution only exists if the minimum of $f(C_{1})$ is less than $E_{1}$. The minimum occurs when $C_{1}={\left(\frac{4w^{2}}{k\mu_{1}}\right)}^{1/3}$ and $f=\frac{3}{2}{\left(\frac{4w^{2}}{k\mu_{1}}\right)}^{1/3}$. Hence, for an active state to exist, we need $k>k_{c}$, where the critical rate is

$$k_{c}=\frac{27w^{2}}{2\mu_{1}E_{1}^{3}}.$$

A discontinuous step transition occurs at the critical point (Figure A1). This step transition is characteristic of reaction systems with second-order autocatalysis, as we have shown [10]. Here, we assumed the external food concentration to be fixed at $E_{1}$ and that there is no catalyst outside the cell. We previously considered cases where external concentrations are variable and where there are additional reactions for spontaneous non-catalyzed synthesis of the catalyst and for direct decay of the food molecule to waste. These things do not change the essential result of a step transition above which an active state is maintained in the cell while the environment is inactive. IO-stability only occurs if the autocatalytic mechanism is second-order in the catalyst concentration. In this case, the inside and outside of the cell can be in different states. In contrast, if the reaction mechanism is first-order, there is only one stable state of the differential equations, and the cell can only be in an active state if the environment is also active. In the context of a protocell, it does not make sense for the metabolism to be occurring in the environment as well as the cell; therefore, first-order autocatalytic systems are not good models for protocells.

### Appendix A.2. Solution for the Homeostatic State

In Figure 3a, we found a homeostatic state in which the internal reaction continues but the size of the vesicle is constant. If the volume is not changing, ∆C must be zero, so all the terms in Equations (2a)–(2g) involving ∆C are zero. If the area is not changing, then $A_{shape}^{+}=A_{lip}^{+}$, $A_{shape}^{-}=A_{lip}^{-}$, and $C_{lip}=E_{lip}=C^{*}$. Now, from (2e), we have $C_{2}=\frac{w}{kC_{1}^{2}}$ (or else $C_{2}=0$), and from (2f), we have $C_{W}=\frac{2w}{\mu_{W}}C_{2}=\frac{2w^{2}}{k\mu_{W}C_{1}^{2}}$. From (2d) and (2e) together, we have $\mu_{1}s\left(E_{1}-C_{1}\right)-2vC_{2}=0$, which can be written as

$$E_{1}=C_{1}+\frac{2w^{2}}{\mu_{1}skC_{1}^{2}}.$$

Using the fact that ∆C=0, we have $E_{1}=C_{1}+C_{2}+C_{W}=C_{1}+\frac{w}{kC_{1}^{2}}+\frac{2w^{2}}{\mu_{W}skC_{1}^{2}}$. Equating these two gives $s=\frac{2w\left(\mu_{W}-\mu_{1}\right)}{\mu_{W}\mu_{1}}$. This shows that there is a particular surface area-to-volume ratio that must exist if this stationary solution exists. There is only a positive solution for *s* if $\mu_{W}>\mu_{1}$. This explains why Figure 3a, in which $\mu_{W}={1.5\mu }_{1}$, reaches a stationary state, although there is no stationary state in Figure 3c, in which $\mu_{W}=\mu_{1}$. When the stationary state exists, the volume and area are $\frac{V}{V_{0}}=1/s^{3}$ and $\frac{A}{A_{0}}=1/s^{2}$. For the parameters of Figure 3a, $s=\frac{2}{3},\frac{A}{A_{0}}=\frac{9}{4},\frac{V}{V_{0}}=\frac{27}{8}$, which agrees with the results in the figure.

### Appendix A.3. The Capsule Shape

When the natural area of the lipids in the membrane $A_{lip}$ is greater than the area of the sphere $A_{sph}(V)$ required to enclose the volume V, the membrane is relaxed and the vesicle has an elongated shape with an actual area $A=A_{lip}$. The radius of a sphere that has this area is $R_{sph}={\left(\frac{A_{lip}}{4\pi }\right)}^{1/2}$. The volume of a sphere with this area is $V_{sph}=\frac{4\pi }{3}R_{sph}^{3}=\frac{1}{6\sqrt{\pi }}A_{lip}^{3/2}$. In terms of the standard vesicle volume and area, $\frac{V_{sph}}{V_{0}}={\left(\frac{A_{lip}}{A_{0}}\right)}^{3/2}$. The reduced volume is defined as $v=\frac{V}{V_{sph}}$, which can also be written $v=\frac{(V/V_{0})}{{\left(A_{lip}/A_{0}\right)}^{3/2\;}}$. We treat the shape as a capsule, consisting of two hemispheres with a radius R linked by a cylinder of length L. The values of R and L must satisfy

$$4\pi R^{2}+2\pi RL=A_{lip},\;\frac{4\pi }{3}R^{3}+\pi R^{2}L=V,$$

or equivalently

$$\frac{R^{2}}{R_{sph}^{2}}+\frac{RL}{2R_{sph}^{2}}=1,\;\frac{R^{3}}{R_{sph}^{3}}+\frac{3R^{2}L}{4R_{sph}^{3}}=v.$$

Solving these two equations gives $R/R_{sph}$ and $L/R_{sph}$ as a function of v.

A capsule can divide into two spheres whose sizes depend on v. The reduced volume is $v=V/(\frac{4\pi R_{sph}^{3}}{3})$. Now, suppose the daughter vesicles are spheres with radii of $R_{1}=r_{1}R_{sph}$ and $R_{2}=r_{2}R_{sph}$. Conservation of the area implies $r_{1}^{2}+r_{2}^{2}=1$. Conservation of the volume implies $r_{1}^{3}+r_{2}^{3}=v$. Therefore, to satisfy both constraints, $r_{1}$ must be the solution of $r_{1}^{3}+{\left(1-r_{1}^{2}\right)}^{3/2}=v$. When $r_{1}=r_{2}=1/\sqrt{2}$, both vesicles are of an equal size. This is only possible when $v=1/\sqrt{2}$.

### Appendix A.4. The Area Difference Energy Model

This model has been widely used previously to determine minimum energy shapes of vesicles under various conditions [24,25,26,27,28]. For a surface with a general shape, there are two radii of curvature $R_{1}$ and $R_{2}$ at any point, and the mean curvature is defined as $H=\frac{1}{2}(\frac{1}{R_{1}}+\frac{1}{R_{2}})$. The total energy of the vesicle is written as the sum of the curvature energies and elastic energies: $E_{tot}=E_{curv}+E_{el}$. The mean curvature energy is defined as

$$E_{curv}=\frac{\kappa }{2}\int dA{\left(2H\right)}^{2}$$

where κ is the bending modulus. The curvature energy for a sphere is $E_{curv}=\frac{\kappa }{2}\left(4\pi R^{2}\right)\frac{4}{R^{2}}=8\pi \kappa$. An additional term for Gaussian curvature can also be added, although this is constant when the topology does not change.

The elastic energy of the two leaflets depends on the difference between the actual areas of the leaflets (determined by the shape) and the preferred areas (determined by the number of lipids):

$$E_{el}=\frac{k_{el}}{2}\frac{{\left(A_{shape}^{+}-A_{lip}^{+}\right)}^{2}}{A_{lip}}+\frac{k_{el}}{2}\frac{{\left(A_{shape}^{-}-A_{lip}^{-}\right)}^{2}}{A_{lip}},$$

where k is the elasticity modulus for the area expansion of a monolayer.

The integrated mean curvature over the surface is $M=\int dA\;\frac{1}{2}(\frac{1}{R_{1}}+\frac{1}{R_{2}})$. The shape areas of the outer and inner leaflets are determined at radii R+d and R−d, which are the midpoints of the two leaflets. We can write $A_{shape}^{+}=A+\frac{∆A_{shape}}{2}$ and $A_{shape}^{-}=A-\frac{∆A_{shape}}{2}$, where the shape-dependent area difference is $∆A_{shape}=4dM$.

The mean lipid area of the two leaflets is $A_{lip}=\frac{1}{2}(A_{lip}^{+}+A_{lip}^{-})$, and the difference in lipid areas is $∆A_{lip}=A_{lip}^{+}-A_{lip}^{-}$; hence, $A_{lip}^{+}=A_{lip}+\frac{∆A_{lip}}{2}$ and $A_{lip}^{-}=A_{lip}-\frac{∆A_{lip}}{2}$. Substituting these into the formula for the elastic energy, we can rewrite it as

$$E_{el}=\frac{k_{el}{\left(A-A_{lip}\right)}^{2}}{A_{lip}}+\frac{k_{el}{\left(∆A_{shape}-∆A_{lip}\right)}^{2}}{4A_{lip}}.$$

The first of these terms is the area expansion term. This is zero for a relaxed vesicle but becomes high for a swollen vesicle with $A>A_{lip}$. The second term is the area difference energy, which depends on the shape of the vesicle and the distribution of lipids between the leaflets.

The elasticity modulus $k_{el}$ that appears in $E_{el}$ is related to the bending modulus for the bilayer, κ, $k_{el}=\beta \kappa /d^{2}$, where β is a constant of order 1, as shown by the following argument (see also [24]). For a monolayer that expands from area A to A+δA, the elastic energy is $E_{el}=\frac{k_{el}{\left(\delta A\right)}^{2}}{2A}=\frac{k_{el}}{2}A\varepsilon^{2}$, where the strain is ε=δA/A; $k_{el}$ has units of energy per unit area. If the monolayer has a thickness of 2d and is made of a uniform elastic material, the modulus of the material (energy per unit volume) must be k/2d. Integrating this over the membrane with uniform strain gives

$$E_{el}=\int_{0}^{2d}\frac{1}{2}\left(\frac{k_{el}}{2d}\right)A\varepsilon^{2}dz=\frac{k_{el}}{2}A\varepsilon^{2},$$

as we expect. Now, if the membrane is curved into a sphere of radius R, the area at the midpoint is $A_{0}=4\pi R^{2}$. The area at a distance z from the midpoint is $A\left(z\right)=4\pi R^{2}(1+\frac{2z}{R}\ldots )$. The curvature energy for the bilayer is

$$E_{curv}=\frac{1}{2}\left(\frac{k_{el}}{2d}\right)\int_{-2d}^{2d}4\pi R^{2}{\left(\frac{2z}{R}\right)}^{2}dz=\frac{64\pi }{3}k_{el}d^{2}.$$

However, when written in terms of the bending modulus, we already know that the curvature energy for a sphere is $E_{curv}=8\pi \kappa$. Equating these gives $k_{el}=3\kappa /8d^{2}$, or β=3/8.

Putting all of the energy terms together, the total energy of the membrane is

$$E_{tot}=\frac{\kappa }{2}\int dA{\left(\frac{1}{R_{1}}+\frac{1}{R_{2}}\right)}^{2}+\frac{\beta \kappa {\left(A-A_{lip}\right)}^{2}}{d^{2}A_{lip}}+\frac{\beta \kappa {\left(∆A_{shape}-∆A_{lip}\right)}^{2}}{4d^{2}A_{lip}}\;$$

This equation ignores the Gaussian curvature term because it is constant for shape changes that preserve the topology. For a capsule shape, the mean curvature is H=1/R in the hemispheres, and $H=\frac{1}{2R}$ in the cylinder. Therefore, the curvature energy is

$$E_{curv}=\frac{\kappa }{2}\left(4\pi R^{2}\frac{4}{R^{2}}+2\pi RL\frac{1}{R^{2}}\right)=\pi \kappa (8+\frac{L}{R}).$$

The curvature energy depends only on the ratio L/R, which is a function of v. The shape-dependent area difference is $∆A_{shape}=4dM$.

$$∆A_{shape}=4d\left(4\pi R^{2}\frac{1}{R}+2\pi RL\frac{1}{2R}\right)=16\pi d\left(R+\frac{1}{4}L\right)=16\pi dR_{sph}(\frac{R}{R_{sph}}+\frac{L}{4R_{sph}})$$

The area difference parameter is $\phi =\frac{∆A_{lip}}{∆A_{shape}}$, so the area difference energy can be written as

$$\frac{\beta \kappa {\left(∆A_{shape}-∆A_{lip}\right)}^{2}}{4d^{2}A_{lip}}=\frac{\beta \kappa ∆A_{shape}^{2}}{4d^{2}A_{lip}}{\left(1-\phi \right)}^{2}=16\pi \beta \kappa {\left(\frac{R}{R_{sph}}+\frac{L}{4R_{sph}}\right)}^{2}{\left(1-\phi \right)}^{2}$$

As the ratios $R/R_{sph}$ and $L/R_{sph}$ are simply functions of v, the area difference energy is a function of v and ϕ. In summary, to determine $E_{tot}$ for a given vesicle, we need to specify the volume V and the two lipid areas $A_{lip}^{+}$ and $A_{lip}^{-}$. From this, the reduced volume v and the area difference parameter ϕ can be calculated, and the value of $E_{tot}$ depends on only v and ϕ.

### Appendix A.5. Energy Change During Division

When using the energy decrease rule for division, we attempt to divide the parent vesicle of volume V into daughter vesicles with volumes $V_{1}=xV$ and $V_{2}=\left(1-x\right)V$, where x is chosen randomly between 0 and 1. We allow an attempted division if the change in energy ∆E≤0. This ∆E depends on v and ϕ for the parent vesicle, and on x. It is also necessary to determine the lipid areas $A_{lip-1},\;A_{lip-2}$ for the two daughters from the parent lipid area $A_{lip}$. We assume that vesicle 1 is a relaxed spherical bud with the necessary lipid area for the sphere, and that the remaining area is given to vesicle 2; therefore,

$$\frac{A_{lip-1}}{A_{0}}={\left(\frac{V_{1}}{V_{0}}\right)}^{2/3},\;\frac{A_{lip-2}}{A_{0}}=\frac{A_{lip}}{A_{0}}-\frac{A_{lip-1}}{A_{0}}.$$

This rule for area distribution is asymmetric, as it treats vesicle 1 as a sphere, while any excess membrane area is given to vesicle 2. We also consider a symmetric area division rule below and show that this is less favorable than the asymmetric rule.

Figure A2 shows ∆E as a function of x for two values of v. For each v, three curves are shown for different ϕ values. The middle curve (red) is for $\phi =\phi_{min}(v)$, at which point ∆E=0 for the optimum division ratio $x=x_{opt}$. The upper curve (black) is for $\phi \leq 0.9\phi_{min}(v)$, where ∆E>0 for all x. The lower curve (blue) is for $\phi =1.1\phi_{min}(v)$, where there is a range of x for which ∆E<0. Figure A2a shows the case of v=0.75, which is greater than $\frac{1}{\sqrt{2}}$. In this case, the optimal division is to form two spheres. The radius of vesicle 1 is $r_{1}$ (obtained in Appendix A.3), and the volume fraction is $x_{opt}=r_{1}^{3}/v$. When $x=x_{opt}$, the second vesicle is also a sphere, i.e., $\frac{A_{lip-2}}{A_{0}}={\left(\frac{V_{2}}{V_{0}}\right)}^{2/3}$. There are two points, $x=x_{opt}$ and $x={1-x}_{opt}$, at which ∆E is the minimum. At $\phi =\phi_{min}\left(v\right)$, ∆E=0 at these two points.

Figure A2b shows the case with v=0.6. Here, the area constraint is always satisfied for any choice of x; however, there is still an optimum division ratio $x_{opt}$ at which the ∆E curve just touches the zero line. The calculated function $\phi_{min}(v)$ is shown in Figure 2a, and the value of $x_{opt}$ is shown as a function of v in Figure 2b. Although the model predicts the position of the division boundary for $v<\frac{1}{\sqrt{2}}$, in fact we find that the trajectories of the vesicles never cross the division boundary when $v<\frac{1}{\sqrt{2}}$ (see Figure 7b and Figure 8).

The division boundary line also depends on the asymmetric area distribution rule used above, which assumed that vesicle 1 was a sphere and that the excess area was assigned to vesicle 2. We also considered a symmetric area distribution rule, in which the excess area is distributed evenly. After assigning volumes, $V_{1}=xV$ and $V_{2}=\left(1-x\right)V$, the minimum possible area for vesicle 1 (when vesicle 1 is a sphere) is $\frac{A_{lip-1}}{A_{0}}={\left(\frac{V_{1}}{V_{0}}\right)}^{2/3}$. The maximum possible area for vesicle 1 (when vesicle 2 is a sphere) is $\frac{A_{lip-1}}{A_{0}}={\frac{A_{lip}}{A_{0}}-\left(\frac{V_{2}}{V_{0}}\right)}^{2/3}$. The midpoint of the minimum and maximum areas is $\frac{A_{lip-1}}{A_{0}}={\frac{A_{lip}}{A_{0}}+\frac{1}{2}{\left(\frac{V_{1}}{V_{0}}\right)}^{2/3}-\frac{1}{2}\left(\frac{V_{2}}{V_{0}}\right)}^{2/3}$. In this case, $\frac{A_{lip-2}}{A_{0}}={\frac{A_{lip}}{A_{0}}+\frac{1}{2}{\left(\frac{V_{2}}{V_{0}}\right)}^{2/3}-\frac{1}{2}\left(\frac{1}{V_{0}}\right)}^{2/3}$, in which case both daughter vesicles are capsule shapes. We calculated $\phi_{min}(v)$ again, with the areas set to the midpoint values. If $v\geq \frac{1}{\sqrt{2}}$, the optimum division is to form two spheres, in which case $\phi_{min}(v)$ is the same for the midpoint and the asymmetric area rules. If $v<\frac{1}{\sqrt{2}}$, the manner of distributing the area makes a difference, although $\phi_{min}(v)$ for the midpoint rule is higher than for the asymmetric rule. Hence, we used the asymmetric rule in all cases.

So far, we have ignored the Gaussian curvature $\kappa_{G}$ in the calculation of ∆E. This is valid up to the point where a single vesicle produces a bud with a narrow neck. Thus, the point ∆E=0 is the point where it is favorable to form a bud with a narrow neck. If the neck is broken to form two separate vesicles, there is an additional contribution to the curvature energy and the change in energy becomes $∆E+4\pi \kappa_{G}$. It is known that $\kappa_{G}$ is negative for many real lipids [59,60,61] with $\frac{\kappa_{G}}{\kappa }\approx -1$. Thus, if we set the division criterion as ∆E≤0, the Gaussian curvature term is always favorable when the neck is broken. Alternatively, we could argue that division is favorable when $∆E+4\pi \kappa_{G}\leq 0$. If we repeat the calculation of $\phi_{min}(v)$ using this criterion, the division boundary line is shifted downwards (to lower ϕ) from that shown in Figure 2a, making division slightly easier to occur. However, if we do this, then the state with the bud connected by a narrow neck would have positive energy with respect to the single vesicle, so the narrow neck would not form spontaneously at this point, and vesicle division would require passing over an energy barrier. Therefore, we argue that setting the division criterion as ∆E≤0 and simply ignoring the Gaussian curvature, as we have done in this paper, is a more reasonable prediction of the point at which spontaneous division will occur.
